# A Review on Graphene-Based Nanomaterials in Biomedical Applications and Risks in Environment and Health

**DOI:** 10.1007/s40820-018-0206-4

**Published:** 2018-05-21

**Authors:** Thabitha P. Dasari Shareena, Danielle McShan, Asok K. Dasmahapatra, Paul B. Tchounwou

**Affiliations:** RCMI Center for Environmental Health, Jackson State University, Jackson, MS 39217 USA

**Keywords:** Graphene-based nanomaterials, Biomedical, Delivery, Biosensors, Tissue engineering, Bioimaging, Health and environment risks

## Abstract

Graphene-based nanomaterials (GBNs) have attracted increasing interests of the scientific community due to their unique physicochemical properties and their applications in biotechnology, biomedicine, bioengineering, disease diagnosis and therapy. Although a large amount of researches have been conducted on these novel nanomaterials, limited comprehensive reviews are published on their biomedical applications and potential environmental and human health effects. The present research aimed at addressing this knowledge gap by examining and discussing: (1) the history, synthesis, structural properties and recent developments of GBNs for biomedical applications; (2) GBNs uses as therapeutics, drug/gene delivery and antibacterial materials; (3) GBNs applications in tissue engineering and in research as biosensors and bioimaging materials; and (4) GBNs potential environmental effects and human health risks. It also discussed the perspectives and challenges associated with the biomedical applications of GBNs.
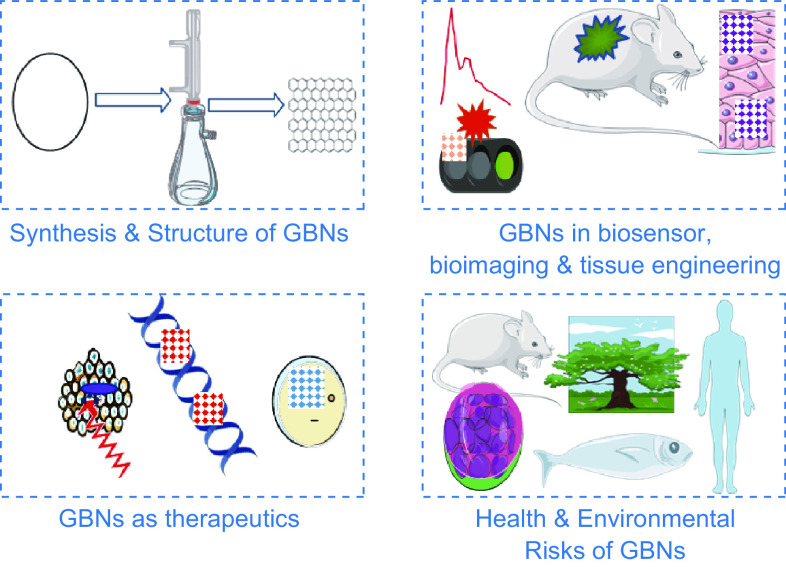

## Highlights

- Structures and properties of graphene-based nanomaterials (GBNs) including graphene, graphene oxide (GO), reduced graphene oxide (RGO) and modified graphene are introduced briefly.
- Recent advances in GBNs for the biomedical applications in drug delivery, biosensor, bioimaging and tissue engineering are summarized and analyzed.
- Potential risks resulted from the vast production and applications of GBNs to the environment and health are discussed to ensure sustainable development of GBNs.

## Introduction

This review focuses on the recent advances in graphene-based nanomaterials (GBNs) in the field of biomedical applications and their potential environmental and health risks. Graphene, the mother of all carbon atoms, is a single atomic thick, nanosized, two-dimensional structure and provides high surface area with adjustable surface chemistry to form hybrids. It was synthesized from graphite. In this review, we addressed the current state of the science and identified the knowledge gap for the future research development. The broad family of GBNs listed in this review includes graphene, graphene oxide (GO), reduced graphene oxide (RGO) and chemically modified graphene (that bears functional groups covalently bound to the surface of the individual layers of graphitic carbon) [1].

### History of GBNs

**Fig. 1:**
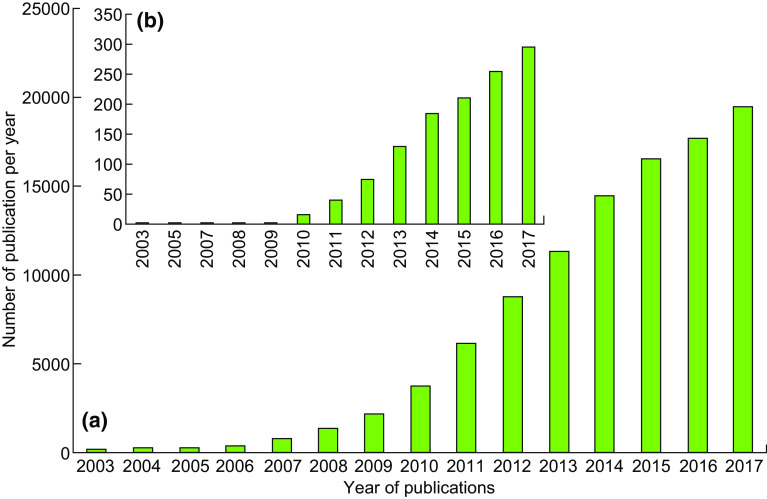
**a** Graphical analysis of a number of publications on ‘graphene’ and **b** ‘biomedical applications of graphene’ for the past 14 years. Data retrieved from Scopus

Although carbon-based materials such as fullerene, graphite, graphene and carbon nanotubes have been widely used due to their unique properties and nanoscale dimensions [2–7], GBNs have attracted considerable interests in recent years (2003–2018) owing to their applications in medicine, biotechnology and various interdisciplinary sciences [8–15]. To date, although significant advances have been made, further studies are needed in many areas related to the multiple biomedical applications of GBNs. A graphical analysis (Fig. 1) of a number of publications was obtained from the years 2003–2017 based on the keywords ‘graphene’ and ‘biomedical applications of graphene’ using Scopus as a search engine. A growing number of publications (Fig. 1a, b) indicate new potential applications of GBNs to anticipate more emphasis on the research with these novel materials. Among GBNs, GO is one of the most potential materials for biomedical applications [16–18]. GBNs, compared to the other carbon-based materials, have the large surface area, easily modified by different functional groups and better solubility that makes them an excellent choice for biomedical use. GBNs are not homogeneous, and they vary in number, lateral dimension, surface chemistry, defect density or quality of the individual graphene sheets and composition or purity [19].

**Fig. 2:**
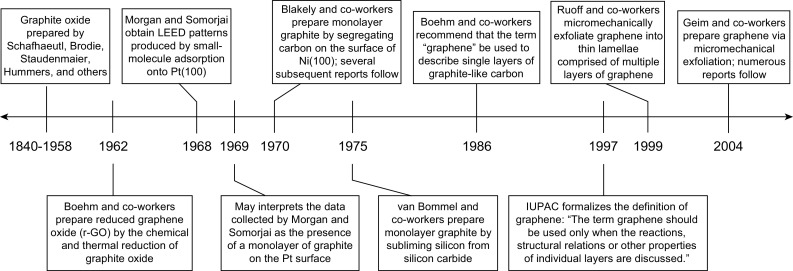
Timeline of selected events in the history of the preparation, isolation and characterization of graphene (Figures are adapted with permission from Ref. [1]). Copyright © 2010 WILEY-VCH Verlag GmbH and Co. KGaA, Weinheim

Even though graphene came into existence in the year 1859 by a British Chemist Benjamin Collins Brodie [20], it has been studied theoretically for many years by Wallace [21]. However, graphene has attracted attention among the scientific community since it was developed as a single layer of material by Novoselov et al. [22] by using the scotch tape method. The timeline of selected events in the history of graphene is highlighted (Fig. 2) [1, 23].

### Structure and Properties of GBNs

**Fig. 3:**
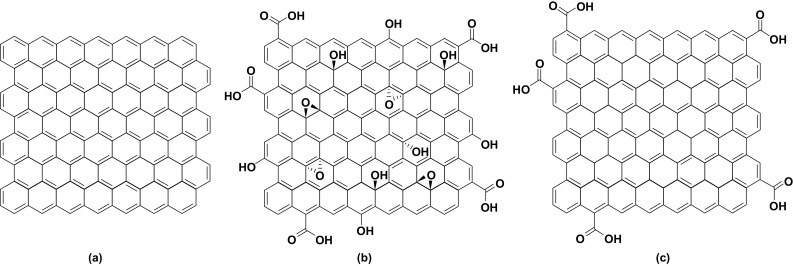
Schematic representation of structures of graphene, GO and RGO [24]. Copyright © Elsevier 2016

GBNs have been classified based on the number of layers in the sheet, oxygen content and their chemical composition. There are many structural differences between GO and RGO which determine their physicochemical properties. Figure 3 is a schematic representation of chemical structures of graphene, GO and RGO [24]. The analytical techniques such as Raman spectroscopy, transmission electron microscopy (TEM), solid-state Fourier transform nuclear magnetic resonance (FT-NMR) spectroscopy and atomic force microscopy (AFM) are being used to understand the structural properties of GBNs [25].

#### Graphene

Graphene is a single carbon layer of the graphite structure. It is a two-dimensional planar and hexagonal array of carbon atoms. Each of these carbons is *sp*^2^-hybridized and has four bonds, one *σ* bond with each of its three neighbors and one *π*-bond that is oriented out of the plane. It has a hexagonal pattern, forming a honeycomb crystal lattice. It is produced by mechanical or chemical exfoliation of graphite via chemical vapor deposition. It has a large specific surface area, high intrinsic mobility and high thermal conductivity. Graphene is considered as hydrophobic because of the absence of oxygen groups.

#### Graphene Oxide

GO is a single layer of graphite oxide, often produced by exfoliation of graphite oxide. GO is produced by acid–base treatment of graphite oxide followed by sonication. Several functional groups such as oxygen, epoxide groups, and carbonyl, hydroxyl and phenol groups are present on the surface of GO. The apparent difference between graphene and GO is the presence of oxygen atoms bound to carbon. GO is the product of hydrophilic derivative of graphene. GO has both aromatic (*sp*^2^) and aliphatic (*sp*^3^) domains which facilitate the interactions at the surface [26–28]. It is synthesized by the Hummer’s method and has oxygenated groups on the surface of the molecule. There is no specific structure for GO, but morphological and structural characterization gives an idea of the GO structure [29].

#### Reduced Graphene Oxide

RGO is the product of graphene oxide or graphite oxide by the chemical or thermal reduction. RGO is considered as an intermediate structure between the ideal graphene sheet and highly-oxidized GO [29]. In addition to the above structural properties of GBNs (Fig. 3), the summary of physicochemical properties of GBNs is listed (Table 1).

**Table 1:** Physicochemical properties of GBNs [118]. Copyright© Elsevier 2014

Property	Single-layer graphene	Graphene oxide (GO)	Reduced GO
Young’s modulus	1000 GPa	220 GPa	N/A
Fracture strength	130 GPa	120 MPa	N/A
Optical transmittance	97.7%	N/A (expected to be lower due to functional groups and defects)	60–90% depending on the reduction agent and fabrication method
Charge carrier concentration	1.4 × 10^13^ cm^−2^	N/A (much lower due to more organic nature, functional groups and defects)	N/A
Room temperature mobility	~ 200,000 cm^2^ V^−1^ s^−1^	N/A (expected to be much lower than 15,000 due to interruption in mobility by defects scattering)	N/A (expected to be intermediate of two due to less defects)
Thermal conductivity	~ 5000 W mK^−1^	2000 W mK^−1^ for pure 600 W mK^−1^ on Si/SiO_2_ substrate	0.14–0.87 W mK^−1^
Electrical conductivity	10^4^ S cm^−1^	10^− 1^ S cm^−1^	200–35,000 S cm^−1^

*N/A* not available

### Synthesis of GBNs

**Fig. 4:**
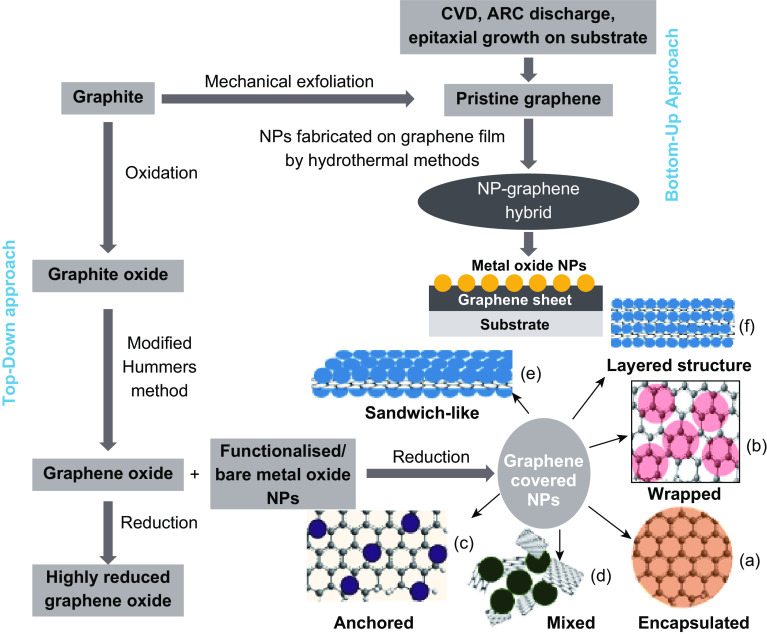
Schematic presentation of graphene synthesis methods—‘top-down’ and ‘bottom-up’—used for the formation of GBN hybrids and different structures. **a** Graphene-encapsulated NPs. **b** Graphene-wrapped NPs. **c** NPs anchored to graphene structures. **d** Mixed graphene-NP structures. **e** Graphene-NP sandwich structures. **f** Graphene-NP layered hybrids [30]. Copyright © 2017 Jana et al.; licensee Beilstein-Institute

Several approaches have been used for the synthesis of GBNs, either a ‘top-down’ or a ‘bottom-up’ approach. Figure 4 illustrates various approaches for the synthesis of GBNs [30]. Each of these methods has its advantages and disadvantages. Reina et al. (2017) emphasized that ‘bottom-up’ method is appropriate to synthesize GBNs rather than ‘top-down’ because of the non-uniformity of the synthesized GBNs which interferes with GBN-based electronic devices for biomedical applications [29]. The size, thickness and the number of layers vary based on the starting material used in the synthesis of graphene [1, 23].

Graphene was synthesized from graphite via mechanical cleavage (Scotch tape method), liquid phase exfoliation, graphite oxide/fluoride reduction, intercalation and compound exfoliation and from non-graphite sources via epitaxial silicon carbide decomposition, chemical vapor deposition (CVD) growth and bottom-up chemical synthesis [31]. Most commonly, GO can be synthesized via Hummer’s method through oxidative exfoliation of graphite using H_2_SO_4_/KMnO_4_ [32]. Moreover, RGO was produced from GO with the use of reducing agents hydrazine, hydrazine hydrate, L-ascorbic acid and sodium borohydride [25]. Additionally, graphene nanocomposites were prepared along with metal and metal oxide nanoparticles via in situ synthetic procedures. These in situ synthetic approaches have concerns such as obtaining uniformity of GO via top-down strategy and control of functional groups on GO, which will affect the quality and properties of GBNs [33]. To better control the size and morphology of the modified GOs, ‘binding method’ is preferred without affecting graphene’s structure. The binding method also has its limitations in size control, binding efficiency, the stability of GBNs and the distance maintenance between fluorescent components of GO and RGO’s [33]. Moreover, functionalization of GO is a vital step to enhance the GBNs for biomedical applications. Covalent and non-covalent approaches facilitate surface functionalization of GBNs to improve solubility, selectivity and biocompatibility [34]. Muthoosamy and Manickam discussed in detail the exfoliation of GBNs and ultrasound-assisted synthesis. Compared to exfoliation, ultrasonication allows synthesis of GBNs in more homogeneous state [23]. Also, Huang et al. listed multiple graphene-NP composites and their applications in various aspects of our daily life [35]. Typically, most of the synthesis approaches involved chemical reducing agents; therefore, researchers have come up with eco-friendly methods using bacteria, phytoextracts and biomolecules during the synthesis just to avoid the hazardous effects of chemical agents [36, 37].

Surface functionalization of GBNs is an essential step to further biomedical applications. Researchers studied to improve the biocompatibility, solubility and selectivity using various polymers and macromolecules such as polyethylene glycol (PEG), polyvinylpyrrolidone (PVP), chitosan, deoxyribonucleic acid (DNA), enzymes and proteins [38].

### Recent Advances of GBNs in Emerging Bioapplications

**Fig. 5:**
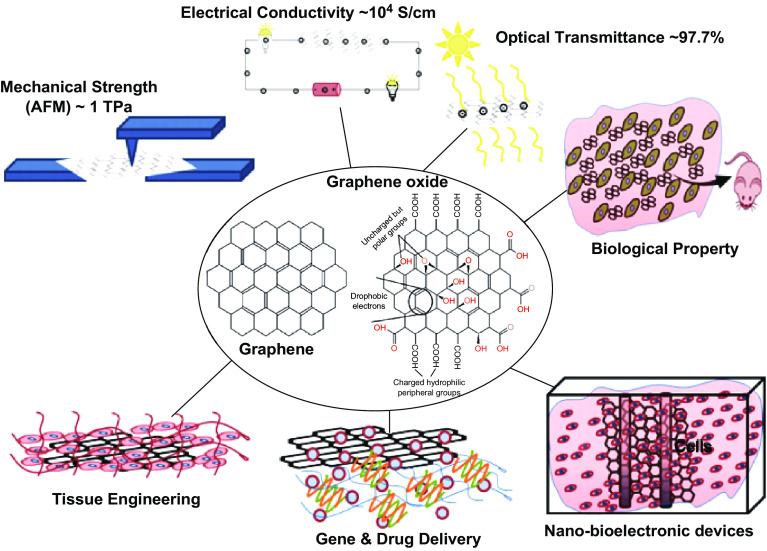
Schematic overview of medical and non-medical applications of GBNs [118]. Copyright © Elsevier 2014

GBNs with their countless applications are expected to revolutionize various areas such as optical, electrical, thermal and mechanical fields (Fig. 5). Mainly, GBNs have received considerable attention for their potential for applications in various areas such as electronics, desalination, metal detection and removal and nuclear waste treatment [19, 39, 40]. Moreover, GO is suitable for biomedical applications such as drug delivery, gene therapy, biomedical imaging, combined cancer therapy, antibacterial agents, as biosensors. However, the actual application of any nanomaterial in biology and medicine is decided critically by its biocompatibility. To date, none of the GO applications have been approved for clinical trials. Some issues related to toxicity and biosafety became pertinent during preliminary biological application of GO [41]. Graphene materials consist of solely carbon. However, it is a matter of serious concern to understand how carbon derivatives like GO and RGO behave in a biological system and how long it takes to excrete from the human body [9]. However, during fabrication, GBNs usually undergo several chemical treatment processes for functionalization, including doping with metals, oxidation, which introduces functional groups, and also a material reduction. This indicates that some of the graphene derivatives considered for bioapplications contain metals and/or impurities other than carbon. It is known from the information on structural properties of GBNs that graphene is a hydrophobic material, so it requires modification of functional groups to make it a biomedical material. This modification may include covalent and non-covalent functionalization. Liu et al. [42] summarized the covalent and non-covalent functionalization. Non-covalent functionalization improves dispersibility, biocompatibility, reactivity, binding capacity or sensing [28]. The formation of hydrogen bonds between polar functional groups on the GO surface and water molecules forms a stable GO colloidal suspension for potential biomedical applications of GO [43, 44]. In bioapplications, both oxidized (GO) and reduced (RGO) graphene oxides are found to be feasible for drug delivery and therapeutic applications. The principal advantage of using GO over other carbon-based materials is due to its aqueous and colloidal stability. The physicochemical characteristics of GO that make it a chemically versatile template with a high surface-to-volume ratio facilitate a variety of biomedical applications such as imaging and cancer therapy, and biosensing. Apart from GO, graphene and RGO have been found to be promising photosensitizing agents for photo-ablation because they generate heat upon irradiation, making it possible for application in combined theranostic therapies.

## Biomedical Applications in Therapeutics of GBNs

Therapeutics is an area of research that deals with the drug delivery and treatment of the infected biological components [45]. During the past 20 years, the rapid development of nanotechnology has brought novel materials which can be used in the diagnosis and therapeutics. Among the carbon nanomaterials, GBNs have gained popularity for their excellent physicochemical properties. Since the discovery of graphene, GBNs are considered as carrier molecules for therapeutics. Properties such as large specific area, *π*–*π* stacking and electrostatic interactions of GBNs facilitate drug loading of partially soluble drugs with high efficiency and potency [46]. GBNs are mostly used in biomedical applications for drug delivery, gene therapy and anticancer therapy.

### Drug Delivery

In the past decade, nanomaterial-based drug delivery systems have been extensively investigated for the treatment of cancer, aiming at improved therapeutic efficacy and reduced toxic side effects. Since 2008, many groups have started to explore graphene-based drug delivery systems. The surface area of graphene (2600 m^2^ g^−1^) is higher, which makes them to be explored for drug delivery [27]. Basically, a monolayer of graphene represents an extreme case where every atom exposed on the surface, which allows significantly higher drug loading capacity. The two prominent modifications reported in the literature for drug delivery using GBNs are chemical modification via electrostatic interaction and binding to the aromatic molecule via *π*–*π* stacking interaction [47, 48]. One more advantage of drug delivery through GBNs is the control of release rate for sustainable drug release [49].

Single-layered GO or RGO has an ultra-high surface area available for highly efficient drug loading [42]. Recently, GO has become quite a competitive drug delivery system with the potential to be applied for systemic targeting and local effective drug delivery [50–52]. GO has unique properties such as surface area, layer number, lateral dimension, surface chemistry and purity which are relevant for their drug delivery and biological applications. In recent years, several studies have been conducted on the delivery of anticancer drugs, genes and peptides through graphene derivatives [7, 19, 24]. Approaches such as simple physisorption via *π*–*π* stacking can be used for loading many hydrophobic drugs, such as doxorubicin and docetaxel, with antibodies for the selective killing of cancer cells. Graphene is a new promising material for drug delivery via the nanocarrier approach, due to its small size, intrinsic optical properties, large specific surface area, low cost and useful non-covalent interactions with aromatic drug molecules. The large specific surface area, *π*–*π* stacking and electrostatic or hydrophobic interactions of graphene can assist in high drug loading of less soluble drugs with high efficiency and potency. Joo and his group reported that PEGylated (covalent conjugation with polyethylene glycol) GO loaded with doxorubicin (DOX) via *π*–*π* interactions shows the promising real-time release of DOX from PEGylated GO at specific loci after an external triggering by glutathione (GSH) [27]. Another research group reported that GO loaded with doxorubicin exhibits higher drug release at pH 5.3 due to the reduced interaction between DOX and the drug carrier [53]. GO loaded with DOX shows enhanced cellular toxicity and promising tumor growth inhibition, with almost 66–91% cell death. Other chemotherapy drugs, such as paclitaxel and methotrexate loaded on GO via *π*–*π* stacking and amide bonds, exhibited an amazing cancerous effect on lung cancer and breast cancer, which resulted in inhibition of about 66–90% of tumor growth. When ibuprofen, which is used as a nonsteroidal anti-inflammatory drug (NSAID), was conjugated with chitosan-functionalized GO joined by amide linkages, the functionalized GO exhibited higher (20%) biocompatibility than GO sheets for human acute lymphoblastic leukemia cell lines (CEM) and Michigan Cancer Foundation 7 cell lines (MCF-7) [54]. GO loaded with a second-generation photosensitizer chlorine 6 (Ce6) resulted in its higher accumulation in tumor cells, leading to a higher photodynamic efficacy upon irradiation. Singh et al. listed various studies on GBNs and their composites used for drug delivery systems [19**].**

It was expected that in 2017, there will be 1, 688,780 new cancer cases diagnosed and 600,920 cancer deaths in the USA [55, 56]. Compared to the normal tissues, tumor tissues usually possess unique microstructural features, unique microenvironment and physicochemical properties such as abnormal temperature gradients, weak acidity, overexpressed proteins and enzymes [57–60]. The altered tumor intracellular environments, such as pH inside of endosomes and lysosomes, are considered when developing the anticancer drug that releases upon reaching the targeted site. For the past two decades, the rapid development in nanotechnology for the diagnosis and treatment of cancer has greatly improved. Among the carbon nanomaterials, GBNs gained popularity in anticancer research. Several studies have contributed to the delivery of GBN-based chemotherapeutics for the treatment of cancer. All great potential of graphene oxide cancer therapies encouraged many researchers to combine multifunctionalities for cancer treatment. In this section, we have summarized the recent reports on the various anticancer drugs used as therapeutics along with GBNs. Also, we have discussed the PDT and PTT used along with GBNs in anticancer therapy. Several studies reported on the delivery of anticancer drugs along with the combination of PTT and PDT. Shim et al. [24] provided a list of few anticancer therapeutics delivered using graphene nanosheets via physical absorption or chemical conjugation. They included doxorubicin, camptothecin, paclitaxel, 1,3-bis (2-chloroethyl)-1-nitrosourea, fluorouracil, methotrexate, lucanthone, β-lapachone and ellagic acid. GBNs loaded with chemotherapeutics are listed in Table 2 [24].

**Table 2:** GBNs loaded with chemotherapeutics [24]. Copyright © Elsevier 2016

Type of GBNs	Chemotherapeutics	Efficacy test	Refs.
GO	Doxorubicin	Doxorubicin-resistant MCF-7	[162]
		CNE1 cells	[163]
GO with PEG	Doxorubicin	Doxorubicin-resistant MCF-7 cells	[164]
	Doxorubicin	HeLa cells	[165]
Citraconic anhydride-functionalized poly(allylamine)/polyethyleneimine-GO	Doxorubicin	U87MG, MCF-7 cells	[166]
Gold nanocluster-decorated RGO	Doxorubicin	HepG2 cells	[167]
Poly(*N*-isopropylacrylamide)-GO	Camptothecin	A-5RT3 cells	[168]
Poly(*N*-vinyl caprolactam)-grafted GO	Camptothecin	KB cells	[169]
Poly(vinyl alcohol)-functionalized GO	Camptothecin	MDA-MB-231 cells	[170]
Starch–graphene nanosheets	Hydroxycamptothecin	SW-620 cells	[171]
Folic acid-modified GO	Doxorubicin, camptothecin	MCF-7 cells	[61]
Poly(lactide) PEG	Paclitaxel	A549 cells	[172]
PEGylated GO	Cisplatin analog	4 T1-bearing mice	[173]
Fe_3_O_4_/graphene nanosheets	Fluorouracil	HepG2 cells	[173]
Chitosan-functionalized GO	Fluorouracil	MCF-7 cells	[54]
Gelatin-functionalized graphene nanosheets	Methotrexate	A549 cells	[174]
Polyacrylic acid-functionalized GO	1,3-Bis (2-chloroethyl)-1-nitrosourea	GL261 cells	[175]
Graphene with PEG	Lucanthone	U251 cells	[50]
Fe_3_O_4_/RGO, Fe_3_O_4_/GO	β-Lapachone	MCF-7 cells	[176]
RGO (modified nanoprobe)	β-Lapachone	MCF-7 cells	[177]
Poloxamer 108-GO, Tween 80-GO, Maltodextrin-GO	Ellagic acid	MCF-7, HT-29 cells	[178]

*N/A* not available; the numbers in the parentheses are respective references

Shim et al. [24] listed various anticancer drug categories used in combination with GBN derivatives. These include anthracycline antibiotics, quinolone alkaloids, taxanes, platinum complexes, nitrosourea compounds, pyrimidine analogs, polyphenolic compounds, quinone compounds and other chemotherapeutics [24]. Zhang et al. established a simple strategy to synthesize a 3D nanoscaled, biocompatible, reduction-responsive nanocarrier [(GON–Cy-ALG–PEG), which is used to deliver anticancer drug DOX with high loading and triggered the release of DOX. They could achieve combined chemo- and photo-thermal therapy better than routine therapy [61]. The multifunctional nanocomposite could make the specific treatment and early diagnosis of different tumors a reality.

Chemotherapy and radiation therapy are major therapeutic approaches for the treatment of a wide variety of invasive cancers today. However, one of the major disadvantages of chemotherapy and radiotherapy is their limited specificity to cancer cells, which lead to the obliteration and other damages to normal tissues and organs. Light irradiation therapeutics, including PTT and PDT, are currently the most promising technology approved by Food and Drug Administration (FDA) to attack cancer with reduced systemic toxicity and improvement of anticancer therapy [62, 63]. Sreejith et al. described schematic illustrations of PTT and PDT approaches [15]. Chen et al. outlined the recent progress in PTT-related applications of GO [64]. The intrinsic optical absorbance of GBNs in the near-infrared (NIR) region contributes to photo-thermal therapeutic use [65, 66].

The efforts to develop suitable phototherapeutic nanomaterial-targeted cancer cells or tumor are in progress [67–70]. In the past few years, phototherapies based on the unique optical and chemical properties of graphene have raised interest. Compared to noble metal nanoparticles and carbon nanotube, graphene materials, especially GO, possess excellent properties such as greater optical absorption in the NIR region and higher photo-thermal conversion, high specific area and lower cost [71–74]. This makes GBNs an ideal candidate for phototherapy. Within the past few years, the strong intrinsic optical absorbance of GO-hybrid materials has been intensively studied for their promising applications in *in vivo* cancer phototherapy [74–78]. PTT employs an optical-absorbing agent to generate heat under light irradiation, so biological tissues are exposed to a raised temperature to promote the selective destruction of abnormal cells. GO has attention from the PTT field due to its strong optical absorption in the near-infrared reflectance region.

Zhang et al. developed a dual-drug-loaded, doxorubicin (DOX)-loaded PEGylated nanographene oxide (NGO–PEG (polyethylene glycol)–DOX), which can deliver heat and the drug to the tumorigenic region to facilitate the combining chemotherapy and photo-thermal treatment in one system [63]. In vivo results demonstrated that the approach was superior to chemotherapy or photo-thermal treatment alone. Yang et al. constructed an iron oxide nanoparticle (CRGO-IONP) nanocomposite probe to combine the capability of tumor bioimaging with PTT [47]. Under the guidance of magnetic resonance imaging (MRI), this group found irradiation effectively ablated solid tumors with an 808-nm NIR laser at a low power density of 0.5 W cm^2^ with the treatment of RGO–IONP–PEG. Hu et al. synthesized a quantum-dot-tagged CRGO (QD–CRGO) nanocomposite that combines the capability of cell/tumor bright fluorescence bioimaging with photo-thermal therapy [79]. The composite mitigated the toxicity of QDs and prevented fluorescence quenching by maintaining a precisely controlled spacer between the QDs and the RGO. With a folic acid attachment, the composite could target MCF-7 cells selectively. After irradiation at 808 nm, cells were killed by the generated heat from the QD–RGO. The increased temperature also caused a marked decrease in the QD brightness, which provided a means for in situ heat/temperature sensing and an indicator of the progress of the photo-thermal therapy. Just recently, the Chung group has developed protein-functionalized RGO nanosheets of great stimuli-responsive drug delivery, controlled release and photo-thermal enhancement capability [18].

The doxorubicin (DOX)-loaded bovine serum albumin (BSA)-functionalized RGO (DOX–BSA–RGO) nanosheets demonstrated NIR-induced chemo-photo-thermal therapy of brain tumor cells treated with DOX–BSA–RGO nanosheets without causing a cytotoxic effect before cell intake. Unlike PTT, PDT relies on irradiation of photosensitizers (PSs) with a suitable light to generate free radicals, which results in irreversible damage to cancer cells. However, PDT is still a challenging technique because many of the commonly used PSs are hydrophobic and cause solubility and biocompatibility problems [45]. In efforts to this issue, Zhou et al. immobilized hypocrellin A (HA, a perylene quinonoid hydrophobic non-porphyrin photodynamic antitumor drug) onto GO via the *π*–*π* stacking interaction, hydrophobic effect and hydrogen-bonding interactions [63]. GO–HA nanomaterial could be excited by irradiation with light of an appropriate wavelength to generate singlet oxygen. The in vitro tests with HeLa cells revealed highly efficient cellular uptake of GO–HA, and the light irradiation of impregnated cells resulted in significant cell death.

To explore dual benefits of PDT and PTT, Tian et al. loaded chlorine 6 (Ce6), a photosensitizer molecule, on PEG-functionalized GO and delivered the multifaceted, complex nanosheet to KB (HeLa derivative) cells. Results show the low power density of 808-nm laser would promote the delivery of Ce6 molecules by mild local heating because of the photo-thermal effect of GO nanosheets. This is compared with Ce6 or GO–PEG–Ce6 complex without the near-infrared laser, and PDT efficacy against cancer cells was significantly enhanced [80].

Another study by Yang et al. [47] synthesized multifunctional nanocomposite GO–PEG–FA/Gd/DOX to obtain MRI and therapeutic effect. Another report on the combined chemo-photo-thermal therapy by Xu et al. [81] showed low toxic nanocomposites NGOHA–AuNRs–DOX which exhibited 1.5- and 4-fold higher cell death than separate chemotherapy and photo-thermal therapy, with biosafety and low side effects compared to non-targeting cells. Au nanoribbon (AuNR)–PEG–GO nanocomposites tested in both in vitro and in vivo showed effective chemo-photo-thermal therapy. An ideal nanocomposite combining GO with gold nanoribbon, AuNR–PEG–GO, was synthesized and used for PTT due to AuNR and GO possessing a strong NIR absorption. The composite properties of AuNR–PEG–GO would also be helpful for introducing appropriate functional groups to target specific cancer cells. The AuNR surfaces could also be a good platform through which proteins and other molecules could be linked to target specific cancer cells after inserting the appropriate cross-linkers [82].

Wang et al. developed chitosan (CS)-modified graphene nanogel for noninvasive controlled drug release. In their study, a NIR-triggered drug delivery platform based on the CS-modified chemically reduced graphene oxide (CRGO) incorporated into a thermo-sensitive nanogel (CGN) was developed. The poly(N-isopropyl acrylamide) (PNIPAM) underwent a reversible discontinuous phase transition in water, changing from hydrophilic to hydrophobic, in response to temperature change. This proved that PNIPAM hydrogel was a thermo-sensitive material. CGN exhibited a NIR-induced thermal effect similar to that of CRGO, reversible thermo-responsive characteristics at 37–42 °C and high DOX loading capacity (48 wt%). The DOX-loaded CGN (DOX–CGN) released DOX faster at 42 °C than at 37 °C. When incubated at 37 and 42 °C, DOX–CGN expression was observed in the cytoplasm of cancer cells, and nucleus, respectively, which was revealed thorough fluorescence images. Upon irradiation with NIR light (808 nm), a rapid, repetitive DOX release from the DOX–CGN was observed. Furthermore, the cancer cells incubated with DOX–CGN and irradiated with NIR light displayed significantly greater cytotoxicity than without irradiation owing to a NIR-triggered increase in temperature leading to nuclear DOX release. These results demonstrated that CGN’s promising application for on-demand drug release by NIR light is very promising [83].

Jinet al. fabricated GO-modified polylactic acid (PLA) (GO–PLA) microcapsules containing AuNPs and used them for ultrasonic (US)/computed tomography (CT) bimodal imaging-guided PTT. After the use of the microcapsules, the US/CT imaging could offer the accurate size and location of the tumor under the real-time guidance and monitoring, and then the NIR laser-induced PTT could be carried out by the diagnostic imaging results without compromising the normal tissues. This was a promising method suitable for tumor therapy [84].

Recently, a pH-responsive nanocarrier by coating nanographene oxide (NGO) with dual types of polymers, PEG and poly(allyl amine hydrochloride) (PAH), was synthesized; the PAH was then modified with 2,3-dimethylmaleic anhydride (DA) to obtain pH-dependent charge reversibility. Moreover, a chemotherapy drug (DOX) was loaded on it; this acquired NGO–PEG–DA/DOX complex exhibited a dual pH-responsiveness, showing distinctly improved cellular uptake under the tumor microenvironmental pH and augmented DOX release under lowered pH inside cell lysosomes. Combining such a unique behavior with the followed slow efflux of DOX, NGO–PEG–DA/DOX offered remarkably enhanced killing of drug-resistant cancer cells under the tumor microenvironmental pH in contrast to free DOX. The combined chemical therapy and PTT were then achieved using NGO–PEG–DA/DOX complex, realizing a synergistic therapeutic effect. This work presented a novel design of surface chemistry on NGO for the development of smart DDSs responding to the tumor microenvironment such as pH with the potential to overcome drug resistance [85].

Multimodality therapy and theranostics are going to attract great attention worldwide owing to its controllable release, minimally invasive properties and high therapeutic efficacy. The multifunctional nanocomposite shows either high photo-thermal energy conversion coefficient or NIR-triggered drug release or pH-sensitive properties or targeting properties with the real-time imaging guidance. So, the construction of other NGO-encapsulated functional nanomaterials for synergistic therapy of malignancy deserves our further efforts. Some most recent examples of multimodality therapy and theranostics are shown by Nellore et al. [86]. Their study investigated the highly selective detection of tumor cells from infected blood samples using AGE-aptamer-conjugated theranostic magnetic nanoparticle-attached hybrid graphene oxide. Their experimental data indicate that hybrid graphene can be used as a multicolor luminescence platform for selective imaging of G361 human malignant melanoma cancer cells. The reported results have also shown that indocyanine green (ICG)-bound AGE-aptamer-attached hybrid graphene oxide is capable of combined synergistic photo-thermal and photodynamic treatment of cancer. Targeted combined treatment using 785 nm NIR light indicates that the multimodal therapeutic treatment is highly effective for malignant melanoma cancer therapy. Hu et al. constructed a photo-theranostic nanoagent using indocyanine green-loaded polydopamine-reduced graphene oxide nanocomposites (ICG–PDA–RGO) and determined if the nanostructure could have amplifying PA and PTT effects for cancer theranostics. The results demonstrate that the PDA layer coating on the surface of RGO could effectively absorb a large number of ICG molecules, quench ICG’s fluorescence, and enhance the PDA–RGO’s optical absorption at 780 nm. The obtained ICG–PDA–RGO exhibits stronger PTT effect and higher PA contrast than that of pure GO and PDA–RGO. After PA imaging-guided PTT treatments, the tumors in 4T1 breast subcutaneous and orthotopic mice models are suppressed completely and no treatment-induced toxicity is observed [87].

### Gene Delivery

GBNs can interact not only with the drugs, but also with other biomolecules like nucleic acids, DNA and RNA. Thus, they can be used as carriers and in the identification of nucleic acids due to large *sp*^2^-hybridized carbon area [88]. Recently, gene therapy has become an important method for treating diseases in regenerative medicine. GO has been demonstrated to adsorb nucleobases by *π*–*π* interaction and also efficiently protect nucleotides from enzymatic cleavage. The basic requirements of a gene delivery vector include protecting DNA from degradation and ensuring high transfection efficiency. Besides, viral and non-viral vectors also have been widely investigated for gene delivery research. Paul et al. [89] found that GO complexed with vascular endothelial growth factor-165 (VEGF) proangiogenic gene is an efficient deliverer for myocardial therapy. Also, graphene oxide nanosheets have been found to be suitable as a vector which is easily up taken by cells [89]. For example, Feng et al. [90] used a polyethylenimine-GO (PEI-GO) carrier to transfect the plasmid DNA into HeLa cells and showed that PEI-GO caused enhancement of the transfection efficiency by proton-sponge effect.

Non-viral gene therapy is a promising approach to treating various diseases caused by genetic disorders [91]. These carriers can transfect cells with new genes from the liquid phase in a conventionally bulky approach or from the surface of the predeposited solid phase in a substrate-mediated manner. The gene vehicle or vector must protect the loaded DNA from degradation by cellular nucleases facilitating its uptake with high transaction efficiency. The major challenge preventing the achievement of these goals is the lack of efficient and non-mutagenic vectors or gene vehicles [89, 92]. Given the unpredictability of viral vectors, many researchers have switched to synthetic vectors composed of liposomes or more recently graphene derivatives. It has been shown that GO derivatives can improve the penetration of siRNA or plasmid DNA (pDNA) into cells protecting DNA from enzyme cleavage [93]. Moreover, the cytotoxicity of cationic polyethylenimine (PEI) is significantly reduced after complexation or conjugation with GO. Also, Li et al. (2002) managed to pattern preconcentrated PEI/pDNA on absorbent GO mediating highly localized and efficient gene delivery. The patterned substrates exhibited excellent biocompatibility and enabled effective gene transfection for various cell lines including stem cells [91]. The distinguishing property of PEI-GO compared to other vehicles is its ability to condense DNA at a low mass ratio (+ 49 mV) and effectively transport pDNA through the cytoplasm to the nucleus. Also, other carbon vectors such as GO/chitosan, GO-PEG and GO/polyamidoamine (PAMAM) can also be used to deliver pDNA and siRNA. Liu et al. showed that graphene oleate PAMAM exhibited good compatibility and greatly improved green fluorescent protein gene transfection efficiency (18.3%) in contrast to ultrasonicated graphene (1.4%) and GO PAMAM without oleic modification (7.0%) [89].

Besides its ability to protect DNA, graphene possesses the unique optical property of absorbing NIR light. Tian et al. showed that localized NIR heating of GO–PEG–Ce6 increased its uptake and efficacy against cancer cells. They attributed the enhanced uptake of GO–PEG–Ce6 to an increase in membrane fluidity upon NIR heating [80]. Moreover, Kim et al. demonstrated that NIR irradiation of functionalized reduced GO can change the membrane integrity of endosomes, thus improving the intracellular lifetime of the drug or gene and their delivery efficacy [94, 95]. Tonelli et al. [7] summarized the graphene-based nanocarriers used for gene delivery.

### Antibacterial Activity

Antibiotic resistance has recently become a significant health problem in the world, as there is an increase in the hospital acquired infection from multidrug-resistant pathogens [96]. However, the overuse of traditional antibiotics has led to the problem of antibiotic resistance. From the past two decades efforts have been made to invent novel drugs to treat multidrug-resistant pathogens including nanoparticles. To overcome resistance to antibiotics, many antibacterial medicines have been developed, such as metal and metal oxide nanoparticles [97]. GBNs were proven to be antibacterial because of their unique physiochemical properties. Researchers developed various GBNs-based nanocomposites via surface modification using biomolecules, polymers and inorganic nanostructures to reduce toxicity and increase their antibacterial efficiency. In this part of the section, we have summarized antibacterial activity of GBNs and their mechanism on antibacterial activity.

The versatility of GBNs and various studies confirm that GBNs could be used as antimicrobial agents [98–110]. GBNs and their nanocomposites were used as antibacterial in many fields such as in controlling microbial pathogens [111], wound dressing [112, 113], tissue engineering [114–116], packaging [117], drug delivery [118] and the purification of water [119]. Table 3 shows a recent review that lists various GBNs and their nanocomposites used as antibacterial agents. The promising applications of GBNs as antibacterial in various fields listed are drug delivery, surface infection, dental fillers, membrane antibiotic fouling, water disinfection and food packaging [120]. There are also a vast number of studies on the antibacterial activity of GO and RGO with other metal and metal oxides. GBNs were evaluated for their antibacterial activity (Table 3). In addition, the synergistic antibacterial activity of GBNs was evaluated along with other metal and metal oxides. For example, GO sheets were hybridized with silver nanoparticles (AgNPs) via one-pot hydrothermal, electrostatics interactions, simple missing chemical deposition, sequential repetitive chemical reductions and supercritical CO_2_. Recently, the contradictory reports on the antibacterial activity of functionalized GBNs have been discussed by Hegab et al. [120].

**Table 3:** Antibacterial efficiency of graphene-based nanomaterials (data adapted from Ref. [120]). Copyright © Elsevier 2016

GBN nanocomposites	Bacterial strains	Incubation and concentration [µg mL^−1^]	Inhibition (%)	Refs.
*Graphene (G)*-*based nanocomposite dispersions*				
Graphene oxide (GO)	*Pseudomona aeruginosa*	2 and 175	100	[102]
RGO	*Escherichia coli*	4 and 10^2^	88	[179]
GO	*E. coli*	2 and 85	98.5	[98]
GO	*E. coli*	2 and 40	69.3	[180]
GO	*E. coli*	2 and 40	97.7	[100]
G-quantum dots	*E. coli, Staphylococcus aureus*	0.25 and 200	80/92	[181]
G-gelatine/silver (Ag) nanoprisms	*E. coli*	24 and 10	99.9	[182]
G-AgNPs	*P. aeruginosa*	0.5 and 5	100	[183]
G-AgNPs	*E. coli*	4 and 10^2^	100	[184]
G-AgNPs	*E. coli*	24 and 10	99.9	[185]
G-AgNPs	*E. coli*/*P. aeruginosa*	24 and N/A	18/26 mm	[186]
G-AgNPs	*S. aureus/B. subtilis*	24 and N/A	100	[187]
G-AgNPs	*E. coli*	0.3 and N/A	20 mm	[188]
G-AgNPs	*E. coli/S. aureus*	24 and 10	100	[189]
G-AgNPs	*E. coli/S. aureus*	4 and 45	100	[190]
G-AgNPs/PDDA (polydiallyldimethyl ammonium chloride)	*E. coli*	24 and 50	100	[191]
G-AgNPs/PEI (polyethyleneimine)	*E. coli/S. aureus*	6 and 958	14.8/20.5	[192]
G-AgNPs/PDA (polydopamine)	*E. coli*	24 and 25	23.7 mm	[193]
G-AgNPs/PAA (poly acrylic acid)	*E. col./S. aureus*	24 and N/A	9.9/11.4 mm	[194]
G-AgNPs/aminophenol AgNPs/aminophenol AgNPs/aminophenol	*E. coli*/*S. aureus*	8 and 500	100	[195]
G-Ag/iron oxide (Fe_3_O_4_)	*E. coli*	24 and N/A	N/A	[196]
G-Ag/titanium oxide (TiO_2_)	*E. coli*	2 and 10^2^	67	[197]
G-Fe_3_O_4_	*E. coli*	2 and 6.6 ×10^5^	91.5	[198]
G-ZnO NPs	*Salmonella typhi*/*E. coli.*	24 and 3 ×10^3^	13/11 mm	[199]
G-ZnO NPs	*Salmonella typhi*/*E. coli.*	12 and 31.25/15.62	100	[200]
G-(MnOx), quantum dots/TiO_2_	*E. coli/S. aureus*	18 and 31.25/15.62	10.9/10.5 mm	[201]
G-MnFe_2_O_4_	*E. coli*	N/A and 10^2^	82	[202]
G-TiO_2_	*E. coli*	0.5 and N/A	99.6	[203]
G-CuNPs/poly-l-lysine PLL	*E. coli*	24 and 50	99	[105]
G-Bi_2_WO_6_	*Mixed culture*	18 and 250	100	[204]
G-Ag/cyclodextrin (CD)	*B. subtilis*	24 and 0.05	N/A	[205]
G-cadmium sulfide (CdS)	*E. coli*	1 and 200	99.9	[206]
G-polyethylene glycol (PEG)/PHGC (polyhexamethylene guanidine hydrochloride]	*E. coli/S. aureus*	1 and N/A	100	[207]
G-chitosan (Cs)	*P. aeruginosa*	24 and 3 × 10^3^	100	[208]
G-dithiothreitol	*E. coli*	4 and 10^2^	86	[179]
G-Sand	*E. coli*	24 and N/A	20 mm	[136]
G-Ramizol	*S. aureus*	20 and N/A	100	[209]
*G-based nanocomposite Surfaces*				
G-AgNPs/polyamide (PA)	*E. coli*	1 and 10^3^	98	[210]
Ag/polyethersulfone (PES) [N/A]	*E. coli, S. aureus, P. aeruginosa*	24 and N/A	N/A	[211]
AgNPs/cellulose acetate (CA)	*E. coli*	2 and N/A	86	[212]
AgNPs/polysulfone (PS)	*E. coli*	18 and N/A	100	[149]
Cs	*S. aureus*	3 and 6 × 10^4^	77	[213]
Polyvinyl alcohol (PVA)/Cs	*E. coli*	24 and 4 × 10^3^	8.6 mm	[214]
PES	*E. coli*	4 and N/A	71	[215]
PA	*E. coli*	1 and N/A	65	[216]
PA	*E. coli*	24 and N/A	65	[217]
Polypropylene (PP)	*E. coli*	12 and	64	[218]
PLL/hyaluronic acid (HA) [105]	*E. coli*	4 and 10^5^	66	[219]
Polyester (PE)/resin[N/A]	*P. aeruginosa*	24 and N/A	15 mm	[220]
PA/PLL [80]	*Mixed culture*	24 and 80	99	[221]
PES	*E. coli*	24 and N/A	74.88	[222]
ZnO NWs	*E. coli*	1 and 10^3^	95	[223]
ZnO NWs	*E. coli*	1 and 99.5 × 10^3^	99.5	[223]
Stainless steel (SS)/RGO	*E. coli*/*S. aureus*	1 and N/A	84/95	[224]
Ti	*E. coli*/*S. aureus*	24 and N/A	68.4/72.9	[225]
TiO_2_	*E. coli*	0.5 and N/A	60	[107]
Cu	*E. coli*/*S. aureus*	24 and N/A	56/34	[226]
Benzalkonium bromide (BKB)	*E. coli/Listeria*	48 and 4 × 10^3^	99.3/91	[227]
G-based hydrogels				
BKB/PDA	*E. coli/Listeria*	48 and 4 × 10^3^	99.3/91	[227]
Agarose	*E. coli*/*S. aureus*	N/A and N/A	100	[228]
Ag	*E. coli*/*S. aureus*	0.5 and 2.5 × 10^3^	100	[112]
Ag/PVA	*E. coli*/*S. aureus*	24 and N/A	100	[229]

*N/A* not available; the numbers in the parentheses are respective references

**Fig. 6:**
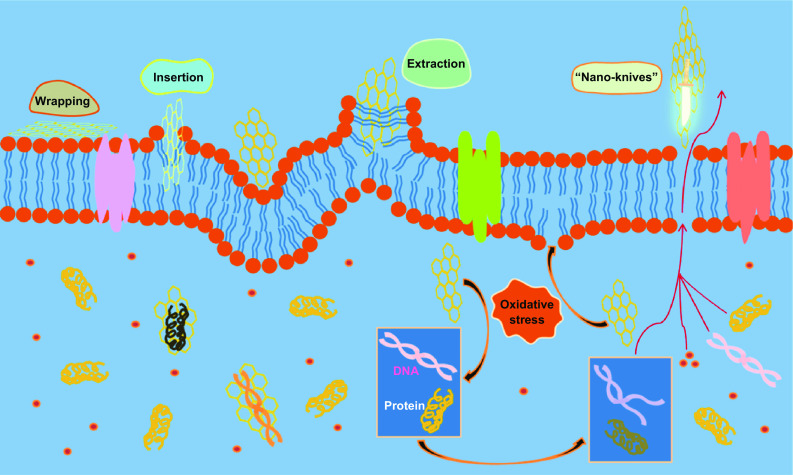
Schematic mechanism of antibacterial activity of GBNs [161]. Copyright © 2016 American Chemical Society

Increasing number of investigations on the antibacterial activity of GBNs postulated several important mechanisms of antibacterial activity [120, 121]. Recently, GBNs have been widely reported to have antibacterial activity with their sharp edges to bacterial membranes leading to the destruction of lipid biomolecules and oxidative stress [121]. Zhao et al. speculated that [68] the antibacterial activity of GBNs is bacterial species dependent rather than gram dependent [122]. The antibacterial activity of GBNs involves several mechanisms together than an individual mechanism responsible for antibacterial activity (Table 3 and Fig. 6). Therefore, it is necessary to compare different types of GBNs and their effects on the bacterial species to their physiochemical characteristics. GBNs physiochemical parameters, impurities from the synthesis process, a method of antibacterial testing and experimental conditions should be considered for the GBNs which are explored for biomedical applications.

From the above discussion, it is evident that GBNs have the potential to be used as therapeutics. GBNs have been loaded with drugs, bacteria, genes and antibacterial agents using various methods based on physisorption, chemical conjugation, gene technology and others. The loading capacity of the GBNs may differ based on the type of GBNs used, nature of drugs, viral and non-viral vectors, and antibacterial materials. Despite the development of GBN-based materials and their applications in chemotherapy, it is essential to focus more on translational research before their use as therapeutics.

## Biomedical Applications of GBNs in Engineering

### Biosensors

Biosensing, bioimaging and therapeutics are three important areas of biomedical research. These three areas are classified based on their functionality. For example, biosensing involves qualitative/quantitative recognition of the specific type of analytes by characterizing the spectrochemical, electrochemical or magneto-chemical behavior of the systems. Mostly, biosensors are useful in the detection of biomolecules and chemical analytes [15]. Biomolecules play a crucial role in the disease development, so the detection of biomolecule aids in the diagnosis and therapy is very important. GBNs can detect these biomolecules due to their excellent electrochemical and optical properties. The capacity to adsorb a variety of aromatic molecules via *π*–*π* stacking interaction makes ideal materials for fabricating biosensors [41]. Biosensors are analytical devices consisting of a biological component (receptor) and electronic component (transducer) [123]. GBNs can be used as biosensors due to their electrochemical and optical properties. They also can adsorb aromatic biomolecules through either *π*–*π* interaction or electrostatic interaction [45]. The functional groups, and electrical and optical properties of GBNs allow them to have the specific interactions at the surface of GBNs. Graphene-based biosensors were developed to detect small molecules such as glucose, nicotinamide, dinucleotide adenine, adenine triphosphate, hydrogen peroxide, estrogen [30, 33, 124–127]. Also GBNs are able to detect macromolecules such as biomarkers to diagnose the disease. The commonly employed techniques such as electrochemical and fluorescence resonance energy transfer (FRET) are being used in the construction of biosensors. The other techniques such as fluorescence spectroscopy, surface plasmon resonance (SPR) and surface-enhanced Raman scattering (SERS) have also shown promising results in the detection of biosensors. Compared to conventional methods, the biosensors are enabled to quantitatively detect small molecules to large biomolecules [68]. Among GBNs, GO exhibits characteristic G-band in Raman spectra along with its water solubility and biocompatibility. Most commonly, GO-based biosensors are capable of lowering detection limits, fast response time, high sensitivity and increased signal-to-noise ratios [128]. GBN nanocomposites work efficiently in combination with metal nanoparticles, auxiliary biomolecules (chitosan), bioenzyme (horseradish peroxidase) due to their enhanced electronic and synergistic compositions to catalyze glucose enzymatic reaction for electrochemical sensing [129–132]. Recent studies have explored on enzyme-based biosensors. The selective and sensitive detection of glucose was reported in the fabrication of GBN-based electrochemical glucose sensors [125, 129, 130, 133]. Other enzyme-based electrochemical biosensors have been developed using enzymes such as horseradish peroxidase (HRP), alcohol dehydrogenase (ADH), organophosphorous hydrolase (OPH), microperoxidase-11, tyrosinase, acetylcholinesterase (AChE), catalase and urease.

### Bioimaging

Bioimaging is considered as the ratification of biosensing outcomes in the detection of the specific type of biological components for diagnostic purposes [45]. Bioimaging is an important aspect of diagnostic research, as it can be used to monitor the health conditions of biological components in typically two types of environments, in vivo and in vitro. The primary requirements of materials used for bioimaging are high specificity, non-toxicity and sensitivity. While graphene can alleviate the toxicity of fabricated probes, introducing the selectivity and sensitivity is still a challenge in the material synthesis. The most widely employed GBNs in bioimaging are graphene quantum dots (GQDs). The initial studies on GQDs as imaging probes were reported in the early 2000s, wherein GQDs were prepared by hydrothermal cutting of graphene sheets [21]. As these dots showed remarkable photo-physical properties, fluorescence spectroscopy was the commonly used technique for imaging biological components. Table 4 outlines various GBNs used for bioimaging.

**Table 4:** GBNs in bioimaging (data adapted from Ref. [33]). Copyright © Elsevier 2016

	Purpose	Advantages	Disadvantages	Studies with GBNS
1 Optical imaging	Utilizes visible light and spectral properties of photons to obtain detailed images of organs and tissues	Low cost, real-time imaging, short acquisition and multiplexing capability	Poor tissue penetrability, strong tissue scattering of photons in the visible light region (395–600 nm)	Nitrogen-doped GQDs [230]
1.1 Fluorescence imaging	Noninvasive technique based on photons emitted from fluorescent probes	Minor auto-fluorescence background, larger imaging depth, reduced photo-bleaching and photo-toxicity	Cannot provide quantitative results. Interference of fluorescence quenching or photo-bleaching of fluorescent dyes, light absorption and scattering or tissues and auto-fluorescence background	nGO-PEG-Rituxan [231]
				nGO–PEG–Cy7 [232]
				GO–IRDye800–VEGF [231]
				GO-PEG [209]
1.2 Two-photon fluorescence imaging (TPMI)	Detailed analysis of cellular/subcellular activities in the deep location of biological samples	This extends the possibility of vibrational spectroscopy with extremely high signal-to-noise ratio, negligible photo-bleaching and multiplexing capabilities to solve chemical and biochemical problems in a nondestructive and non-perturbing manner		N-GQDs [230]
	Excitation wavelength in the range of 700–1350 nm			
1.3 Raman imaging	It exploits the inelastic scattering of phonons derived from molecular vibrational excitation modes			Ag/GO hybrids [210]
				Folic acid-conjugated Ag/GO hybrids [233]
				Au@NGOs [234]
				RGO-NS [235]
				Au/GO and Au/RGO [236, 237]
				AgCu@graphene [238]
2. Radionuclide imaging	Accurately tracks the radiolabeled substances in vivo in a quantitative manner with excellent sensitivity	PET and SPECT imaging		nGO-PEG with 1251 [232]
		Low background signal and require little signal amplification		64 Cu-labeled nGO-PEG [239]
				66 Ga nGO-PEG [240]
				198, 199 Au@AF-GO [241]
3. Magnetic resonance imaging (MRI)	It has been used to image the anatomy as well as function of tissues in a quantitative manner with excellent spatial resolution	Noninvasive technique without ionizing radiation	Low sensitivity, long signal acquisition time	Gd–NGO [242]
				GO–IONP [47]
				RGO–IONP [243]
				Fluorinated GO [244]
4. Photo-acoustic imaging (PAI)	It offers optical absorption contrast with the resolution of ultrasound for deep tissue/organ imaging	Radiofrequency waves exhibit lower scattering in the biological samples		RGO [245]
				ICG–GO [246]
				BSA-nano-RGO [247]
5. Computed tomography (CT)	It provides complementary anatomical information			Go@Ag [248]
	It measures the absorption of X-rays when they pass through targets			GO/BaGdF5/PEG [249]
6. Multimodal imaging	This technique refers to integrating the merits of individual imaging modality and collecting all information from different imaging modalities that offers higher efficiency and accuracy of diagnosis	Avoids the additional stress on the body’s blood clearance that accompanies the administration of multiple doses of agents		RGO–IONP–PEG [243]
				GO–IONP–Au [250]
				GO–BaGdF5–PEG [249]

The numbers in the parentheses are respective references

### Tissue Engineering

Tissue engineering is an emerging new area in life sciences that targets the development of biological substitutes to modify the function of a tissue to repair and maintain its properties. These biological substitutes also known as scaffolds are made of a biodegradable material [134]. Traditional transplantation has limitations to repair tissue damage caused by trauma, infection, tumor and deformity. Materials such as hydrogels lack mechanical strength for cells to attach and spread [135]. However, different tissues in the body possess different mechanical, electrical or physical properties. Single materials might not mimic the physical and biological properties of the native tissue; therefore, hybrid bioactive materials with a variety of components that can address different requirements are widely used to fabricate artificial tissues. Hydroxyapatite [HA; Ca_10_(PO4)_6_(OH)_2_] is commonly used in various forms and shapes in bone and tissue engineering. However, due to their lack of mechanical strength, its usage is discouraged from replacing various parts of the bone system [136]. Materials such as hydrogels and nano-TiO_2_ have been used due to the lack of mechanical strength, aggregation and migration of TiO_2_, limiting their application in tissue engineering [134, 137, 138]. Moreover, the artificial biomarkers such as calcium phosphate (CaP), hydrogels, calcium silicate (Cs) lack the tissue inductive activity and delay the healing of functional modifications. In addition to the above, the compatibility, toxicity and anticoagulant capacity of scaffold material are other factors that limit their use in tissue engineering [137]. We discussed in Section 1.2 of this review, graphene is the basic unit of all forms of GBNs. Graphene has high mechanical strength, high surface area, high conductivity and low density. Graphene is also susceptible to acid and alkali environments and resists corrosion from the surroundings. The unique properties of GBNs, such as high elasticity, flexibility and adaptability to flat and irregular surfaces, make them suitable for the structural reinforcement of materials essential for tissue engineering which can improve adhesion, differentiation and cell function [24, 139, 140]. Among GBNs, GO can be modified easily because of the functional groups such as hydroxyl, epoxy, carboxyl on the surface of GO. Moreover GO, RGO and other graphene-based composites can be easily chemically modified because of the functional groups on the surface to interact with various biological molecules such as DNA, proteins, peptides and enzymes. On the other hand, RGO and other GO composites are being used in tissue engineering due to their flexibility to fabricate. Biomaterials like GO can induce specific cellular functions, direct cell differentiation and modulate cell–cell interactions. The fabrication strategies of graphene with biopolymer, protein, peptide, DNA and polysaccharide were discussed [141]. In the literature, it was reported that the GBNs are also applied in cardiac, neural, bone, cartilage, skeletal muscle and skin/adipose tissue engineering. The reports indicated that GBNs may also have the osteogenic and neural potential [142, 143]. GBNs antimicrobial activity supports its role in tissue engineering by reducing the infections induced by microbes to progress human health [141].

Researchers demonstrated that GO could efficiently support differentiation of stem cells. Park et al. demonstrated that GBNs could be used in stem cell culture substrate to stimulate the cardiomyogenic differentiation process of mesenchymal cells [143]. In another report, Shin et al. developed 3D multilayer tissue constructs and showed strong spontaneous beating and frequency dependency under a low external electric field [114]. The GBN nanocomposite films aid in a suitable environment for the cell growth and the production of extracellular matrix in mesenchymal cells (mMSCs) to differentiated osteoblasts for bone regeneration. Golafshan et al. investigated the cultures of PC12 cells on the scaffolds; the results indicated that these scaffolds could efficiently enhance attachment, spreading and proliferation of PC12 cells [144]. The GBNs ability to maintain high cellular viability for longer periods of time after differentiation is essential for regenerative medicine [141]. In another report, Zhou et al. evaluated GBNs stem cell-based therapies for treating bone diseases [145]. They confirmed that cartilage cells seeded on the GBNs hybrid scaffold retain chondrogenic properties and are suitable substrates. Park et al. solved the problem of the poor survival rate of mesenchymal cells implanted in myocardial tissue by using GO and fibronectin-RGO-MSC hybrids to improve cardiac function restoration [143, 146].

## Health and Environmental Risks of GBNs

GBNs are being used in various biomedical applications in the areas of drug delivery, tissue engineering and antibacterial materials. However, considerable variations need to be addressed before the use of GBNs for treatment in humans as therapeutics. The vast production of GBNs due to their applications might lead to the significant human and environmental exposures. To address the human and environmental risk of GBNs, it is essential to evaluate the level and degree of the toxicity for the effective use of GBNs in biomedical applications [147, 148]. The biological interactions of GBNs can be categorized into biomedical applications and environmental health and safety. Occupational and environmental exposures may also lead to potential toxicity of GBNs through non-biomedical products [149]. It is essential to understand the interactions at the cellular and molecular levels to determine the toxicity of GBNs. By overcoming the challenges to be used in therapeutic delivery, the modification of GBNs in biological systems spurs up further developments in biomedical applications. From a toxicological standpoint, the physicochemical characteristics of GBNs play an essential role in assessing the extent of toxicity. For instance, dose, shape, surface chemistry, exposure route and purity play important roles in differential toxicity of GBNs [19]. Surface area, layer number, lateral dimension, surface chemistry and purity of GBNs also play a vital role in exerting the toxicity [150].

The surface chemistry of GBNs is of utmost importance for any bio-functionalization to be carried out. The surface area of the GBNs decreases as the layer number increases. The number of layers of GBNs is an important characteristic as it determines the specific surface area and bending stiffness. Whereas lateral dimension does not affect specific surface area but defines the dimension of the material, which is significant for the biological phenomena (cell uptake, renal clearance and blood–brain barrier transport) influenced by particle size [150]. Among GBNs, GO is highly reactive because of the solubility and functionalization on the surface compared to graphene and RGO. When it comes to purity, GBNs based on their synthesis process may contain unreacted and residual chemicals resulting in inadequate washing. To report the toxicity in a comprehensive approach, the above-mentioned properties need to be characterized when carrying out biological studies [150]. The route of entry of GBNs into the body via blood circulation or biological barriers may affect different organs. GBNs may enter organs by crossing blood–air barrier, blood testis barrier, blood–brain barrier and blood–placental barrier, because of their nanosize, surface structure, functionalization, charge, impurities, aggregation, corona effects and physical destructions. Several cellular mechanisms such as oxidative stress, DNA damage, inflammatory response, apoptosis, autophagy and necrosis play a significant role in GBNs toxicity [16]. Even though GBNs have suggested various biomedical applications, toxicity and biosafety are the main issues related to their biological applications.

### Toxicity In Vitro and In Vivo

The toxicity of GBNs has been evaluated in different cell lines, including lung epithelial cells, fibroblasts, neuronal cells, cancer cells and animal models (Tables 5, 6). The cell death caused by nanomaterials includes either necrosis triggered by reactive oxygen species or apoptosis via plasma membrane damage. In the past few years, many reviews had published on the toxicity of GBNs in cells and animal models. The review by Ou et al. [16] summarized various toxicity studies conducted in various organs of animals and cells. The data from this review (Tables 5, 6) [16, 38] show the development of biocompatible GBNs and their toxicity effects on the cell and animal models. Moreover, Syama et al. [151] summarized approaches to reduced toxicity of graphene by using a biocompatible GBN, using microbes and plant extracts and biocompatible polymers to produce GBNs.

**Table 5:** GBNs toxicity effects in various cells (data adapted from Ref. [152]). Copyright © Elsevier 2016

GBNs [Exposure conditions]	Cell types	Effects	Refs.
Pristine graphene [20 µg mL^−1^ for 24 h]	Peritoneal macrophages; RAW264.7	Elevated transcription and secretion of cytokines and chemokines, which is triggered by activation of the NF-κB signaling pathway	[251]
Pristine graphene [0-80 µg mL^−1^ for 24 and 48 h]	RAW264.7	Induction of cytotoxicity through the depletion of the mitochondrial membrane potential and the increase in intracellular reactive oxygen species, then trigger apoptosis by activation of the mitochondrial pathway	[252]
Pristine graphene; functionalized graphene [75 g mL^−1^ for 24 or 48 h]	RAW264.7	High intracellular uptake of functionalized, hydrophilic graphene compared to the hydrophobic pristine graphene	[253]
Graphene; few-layer graphene (FLG) microsheets [5 h for macrophages and 24 h for other cell types]	Primary human keratinocytes; human lung epithelial cells; Murine macrophages	GBNs enter cells through spontaneous membrane penetration at edge asperities and corner sites	[254]
Graphene [N/A]	HeLa; Panc-1	The cellular responses are strongly dependent on either cell type or hard corona composition	[255]
Graphene quantum dots (GQDs) [0–200 μg mL^−1^, for 24, 48 or 72 h]	THP-1	Induction of inflammatory response, apoptosis and autophagy in macrophages via p38 MAPK and NF-κB signaling pathways	[256]
Pluronic dispersed graphene; GO (graphene oxide) [administered directly into the lungs of mice]	Lung cells	Increased rate of mitochondrial respiration and the generation of reactive oxygen species, activating inflammatory and apoptotic pathways	[257]
Graphene, GO [20 μg mL^−1^; 24 h]	MDA-MB-231; B16F10	Inhibits the migration and invasion of various cancer cells by inhibiting the activities of ETC complexes	[258]
Carboxyl graphene nanoplatelets (CXYG) [0–32 μg mL^−1^ for 72 h]	HepG2	Cytotoxicity in HepG2 cells with plasma membrane damage and induction of oxidative stress	[259]
GO [1–200 mg L^−1^, 24 h]	HepG2	NADPH oxidase-dependent ROS formation; deregulation of antioxidant/DNA repair/apoptosis-related genes	[260]
GO [100 mg L^−1^ for 48 h]	GLC-82	Alters the miRNA expression profile	[261]
GO [0–16 μg mL^−1^ for 72 h]	HepG2	Caused cytotoxicity in HepG2 cells with plasma membrane damage and induction of oxidative stress	[259]
GO [N/A]	RAW-264.7; Saos-2; 3T3	Impact on cytoskeleton; alterations in cell cycle	[262]
GO and its nanoassemblies [l μg mL^−1^; 24–72 h]	Mouse embryonic fibroblast (MEF)	Without induction of noticeable harmful effects	[263]
GO, bGO, pGO-5, pGO-30 and GS (graphene sheets) [0–200 μg mL^−1^, for 3 or 24 h]	Red blood cells; human skin fibroblasts	All the GO and GS show dose-dependent hemolytic activity on RBCs	[264]
GO [50 μg mL^−1^ for 24 h]	MEF	Higher degree of cytotoxicity and apoptosis.	[265]
GO [0–100 μg mL^−1^ 0–5 days]	Human fibroblast cell	Dose- and time-dependent cytotoxicity, decreasing cell adhesion, inducing cell floating and apoptosis	[266]
GO [N/A]	Red blood cells	Strong hemolytic activity	[267]
GO [20–100 μg mL^−1^ for 0–12 h]	A549	Cytotoxicity of GO is largely attenuated due to the extremely high protein adsorption ability of GO	[268]
GO [0–20 μg mL^−1^]	Peritoneal macrophage; J774A.1; LLC; MCF-7; HepG2; human umbilical vein endothelial cells (HUVEC)	Microsized GO induced much stronger inflammation responses, while nanosized graphene sheet showed better biocompatibility	[269]
GO [5–100 μg mL^−1^ for 24 h]	RAW264.7	Provoked the Toll-like receptor (TLR) signaling cascades and triggered ensuing cytokine responses	[269]
GO [20 μg mL^−1^ for 24 h]	J774A.1; RAW 264.7	Activation of TLR4 signaling leads to GO-mediated macrophagic necrosis	[270]
GO [1–100 μg mL^−1^ for 24 h]	Human monocyte-derived macrophages; peritoneal macrophages	Significant impact on cellular viability, ROS generation and cellular activation	[271]
GO, PVP-GO [25–100 μg mL^−1^ for 48 h]	Dendritic cells	PVP-modified GO has a low immunogenicity than unadorned GO	[272]
GO, TiO_2_-GO [100 and 300 μg mL^−1^ for 4 h]	A549	GO enters A549 cells and locates in the cytoplasm and nucleus without causing any cell damage. The TiO_2_–GO composite separated into GO and TiO_2_ after TiO_2_–GO composite entered A549 cells	[273]
GO, sGO [12.5 μg mL^−1^ for 48 h]	PC-12	Inhibit Aβ peptide monomer fibrillation and clear mature amyloid fibrils	[274]
GO flake [10 μg mL^−1^]	Mesenchymal stem cells (MSC)	GO flakes effectively prevent a series of adverse cell signaling cascades that result in the anoikis of MSCs in response to ROS	[275]
GO [37.5 μg mL^−1^ FITC-PEG-GOs for 2 h]	Saos-2; HepG2; RAW-264.7	Processes such as micropinocytosis, microtubule-dependent mechanisms, clathrin-dependent mechanisms and phagocytosis are involved	[276]
GO [20–50 μg mL^−1^ for 30 min–14 h]	C2C12	Cells enter through clathrin-mediated endocytosis, and the increase in graphene size enhances phagocytotic uptake of the nanosheets	[277]
GO [40 or 80 μg mL^−1^ for 24 h]	MDA-MB-231; MDA-MB-436; SK-BR-3	PEG-GO inhibited the migratory and invasive properties of human metastatic breast cancer cell lines by inhibiting ATP synthesis, leading to a disruption of F-actin cytoskeletal assembly	[278]
NGO [N/A]	HCT-116	No apparent toxicity as drug carrier	[279]
NGO [N/A]	HeLa	No apparent toxicity as drug carrier	[280]
Oxidized graphene nanoribbons (O-GNR) [10–400 μg mL^−1^ for 12–48 h]	HeLa; NIH-3T3; SKBR3; MCF-7	Dose-dependent and time-dependent cytotoxic effects on the four cell lines	[281]
O-GNR [50 μg mL^−1^ for 30 min]	MCF-7; A549; MRC5	Significant O-GNR-PEG-DSPE uptake into cells with high EGFR expression	[282]
O-GNR [N/A]	U251; CG-4; MCF-7	No apparent toxicity as drug carrier	[50]
O-GNR [0–100 μg mL^−1^ for 24 h]	A549	GONRs with concentrations ≤ 50 μg/mL showed no significant cytotoxicity; GONRs with a concentration of 100 μg/mL exhibited significant cytotoxicity and resulted in a decrease in cell growth and induction of cell apoptosis	[283]
O-GNR, GNO and GONP [0–300 μg mL^−1^ 24–72 h]	MSC	GNOs, GONRs and GONPs at concentrations of less than 50 μg/mL for 24 or 72 h could be considered potentially safe incubation conditions for ex vivo labeling for MSCs	[284]
GO; RGO [200 μg mL^−1^ 24 h]	A549	Protein-coated graphene resulted in a markedly less cytotoxicity than uncoated graphene	[285]
GO, RGO [10 μg mL^−1^]	HUVEC	Significant increase in both intercellular ROS levels and mRNA levels of HO1 and TrxR. Moreover, a significant amount of DNA damage is observed in GO-treated cells, but not in RGO-treated cells	[286]
GO, RGO [0.0125–12.5 μg cm^−2^ for 5 days]	A549; RAW 264.7	Lower concentrations of GO/RGO did not lead to an increase in ROS production. Cellular internalization of GO was observed in phago(endo)somes without signs of any intracellular damage.	[287]
RGO/HARGO (hyaluronic acid GO) [20 μg mL^−1^ for 24 h]	KB	No significant cell death observed in the absence of NIR irradiation	[288]
RGO [N/A]	Ramos; CCRF-CEM	No apparent toxicity as drug carrier	[289]
RGO [1–200 mg L^−1^ for 24 h]	HepG2	Hydrophobic RGO was found to mostly adsorbed at cell surface without internalization, ROS generation by physical interaction, poor gene regulation	[260]
RGO [1–100 μg mL^−1^ 24 h]	Human blood cells; HUVEC	The biocompatible biopolymer-functionalized RGO exhibited excellent biocompatibility	[290]
RGO, GONP, RGONP [0.01–100 μg mL^−1^ for 24 h]	MSC	The RGONPs exhibited a strong potential in destruction of the cells with the threshold concentration of 1.0 mg/mL, while the cytotoxicity of the RGO sheets appeared at high concentration of 100 mg/mL after 1 h	[291]
GO, RGO [1–10 μg mL^−1^ for 24 or 48 h]	HUVEC	GO exhibits higher toxicity than RGO due to ROS generation. Small flake size graphene exhibits greater cytotoxicity compared to larger sheets due to intracellular accumulation of graphene	[286]
GO, RGO [0–20 μg mL^−1^]	Human platelets	GO can evoke strong aggregatory response in platelets comparable to that elicited by thrombin	[292]
GO, RGO, G-NH_2_ [2–10 μg mL^−1^ for 3 h]	Red blood cells	G-NH_2_ is not endowed with thrombotoxic property	[293]
GO, RGO [100 μg mL^−1^]	U87 U118	Reduction in cell viability and proliferation and induced apoptosis	[294]
RGO [50 μg mL^−1^]	U87	Reduction in GBM tumor volume was observed. RGO + Arg shows antiangiogenic and proapoptotic characteristics	[295]

The numbers in the parentheses are respective references

**Table 6:** GBNs toxicity effects in various animal models (Data adapted from [16, 296, 297, 298])

GBNs and exposure conditions	Animal model	Effects	Refs.
Nanoscale graphene oxide (NGO) [0, 1, 5, 10 mg kg^−1^, intratracheal instillation 0 h, 24 h, 48 h, 72 h and 1 week]	C57BL/6 mice	Acute lung injury (ALI) and chronic pulmonary fibrosis	[299]
Few-layer graphene (FLG) [0.1, or 1 mg mL^−1^, oral gavage or intratracheal instillation 3 or 28 days]	ICR mice	Intratracheally instilled FLG acute lung injury and pulmonary edema, FLG did not show detectable absorption through the gastrointestinal tract by oral gavage	[300]
Graphene platelets (GPs) [inhalation exposure, 1 day–6 weeks]	Mice	GP caused acute inflammation in lung at 1 day and alleviated inflammation in lung after 6 weeks	[301]
Graphene nanoplatelets (GPs) [50 μg per mouse, pharyngeal aspiration or intrapleural installation, 24 h–7 days]	Female C57BL/6 strain mice	Large GPs were inflammogenic in both the lung and the pleural space	[302]
GO [0.5 or 4 mg m^−3^, inhalation exposure, single 6 h]	Sprague–Dawley rats	The single inhalation exposure to GO induces minimal toxic responses in rat lungs	[303]
GO [1.0 mg kg^−1^, intravenously injected, 24 h]	Male ICR mice	Accumulated mainly in the liver and lungs	[303]
GO [24 mg kg^−1^, tail vein injected, 5 days]	Male and female ICR-strain mice	Did not affect pup numbers, sex ratio, weights, pup survival rates or pup growth, low toxicity for male reproduction	[304]
GO [1,10 mg kg^−1^, intravenous injection 14 days]	Kunming mice	Led to high accumulation, long-time retention, pulmonary edema and granuloma formation	[305]
NGO–PEG [5 mg kg^−1^, tail intravenous injection 10 min-24 h]	Male Kunming mice	NGO–PEG alleviated acute tissue injuries and decreased the early weight loss	[306]
GO, GO–PEG, RGO–PEG, nRGO–PEG [4 mg kg^−1^, intraperitoneal injection 1, 7 and 30 days]	Balb/c mice	Accumulated in the reticuloendothelial system (RES) including liver and spleen over a long time	[48]
GO, graphene quantum dots (GQD) [20 mg kg^−1^ intravenous injection or intraperitoneal injection 14 days]	Balb/c mice	GO appeared toxic and caused death GQD revealed no accumulation in organs and caused low cytotoxicity	[307]
Purified graphene oxide (pGO) [50 μg/animal, intraperitoneal injection 24 h, 7 days]	Female C57Bl/6 mice	Induced moderate inflammation and granuloma formation following	[257]
GO [series concentrations, subcutaneous injection 21 days]	C57BL/6 male mice	The microsize of GO induced much stronger inflammation responses than the nanosized GO	[269]
GO [2 or 20 mg kg^−1^, subcutaneous and intraperitoneal injection]	C57BL/6 J mice	Both GO and a reduction in GO result in immune cell infiltration, uptake and clearance	[308]
RGO-iron oxide nanoparticles (RGO-IONP) [400 μg, subcutaneous injection]	Female Balb/c mice	RGO–IONP can effectively inactivate multiple-drug-resistant bacteria in subcutaneous abscesses	[309]
GO, GO-PEG [100 mg kg^−1^, oral administration; 50 mg kg^−1^, intraperitoneal injection, 1, 7 and 30 days]	Female Balb/c mice	No obvious tissue uptake via oral administration, indicating the rather limited intestinal adsorption of those nanomaterials	[48]
RGO [60 mg kg^−1^, oral gavage, 5 days]	Male C57black/6 mice	RGO affected general locomotor activity, balance and neuromuscular coordination, but showed little change in exploratory, anxiety-like or learning and memory behaviors	[48]
GO [0.76 ± 0.16 − 9.78 ± 0.29 mg m^−3,^6 h/day for 5 days]	Male Sprague–Dawley rats	No significant systemic effects of toxicological importance were observed. Only minimal or unnoticeable GO toxicity in the lungs and other organs	[297]
Graphene [0.12–1.88 mg m^−3^, 6 h/day and 5 days a week].	Male Sprague–Dawley rats	No dose-dependent effects and no distinct lung pathology were observed. This study suggested low toxicity, and a NOAEL of no less than 1.88 mg/m^3^ was recorded for the body weights, bronchoalveolar lavage fluid inflammatory markers and blood biochemical parameters	[298]
Graphene [0.68 ± 0.14–3.86 ± mg m^−3^, 6 h/day and 5 days a week]	Male Sprague–Dawley rats	Minimal toxic effect at the concentrations and time points in this study	[296]

**Fig. 7:**
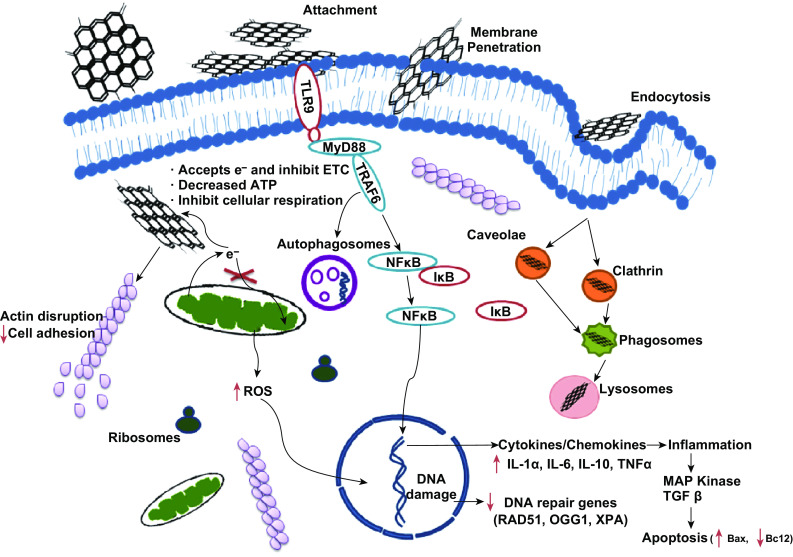
Mechanisms of toxicity of GBNs [151]. Copyright © 2016 Elsevier B.V

The two aspects that demonstrate the behavior of GBNs in biological fluids are the behavior of graphene as a colloid and the formation of the graphene surface of the protein corona. The GBNs in colloid form interact with the physiological media resulting in aggregation and flocculation of the suspension. Another critical factor affecting the behavior of GBNs is the formation of a protein corona. They explain that two components (soft and hard corona) play a significant role in adsorbing proteins. The particle stability may be enhanced if proteins are adsorbed via hydrophobic region to the basal plane of the flake with the hydrophilic region directed toward the exterior. On the other hand, adverse reactions may occur with the biodistribution and the interaction with the immune system. Hence, it is confirmed that the systemic adverse reactions are caused by GBNs or by modifications performed to GBNs [148]. Cells exposed to nanomaterial may undergo both apoptosis and necrosis. Chemical and physical properties such as reactive oxygen species (ROS) and direct damage to plasma membrane may trigger apoptosis and necrosis respectively. Figure 7 illustrates various toxicity mechanisms of toxicity at the cellular level [151]. Many reports were published on the internalization of GBNs as therapeutic agents as well as they might lead to cell intoxication [152]. The complication of intravenous drug delivery of GBNs bioaccumulation and granuloma formation can be overcome by surface modifications to accomplish selective targeting and support biodegradation [150].

It is evident from the literature [16] that in vitro toxicity results suggest that GBNs can be mostly toxic, but the toxicity is dependent on various factors such layer number, lateral size, stiffness, hydrophobicity, surface functionalization and dose. The four routes for entry of any nanoparticle into the human body include inhalation, ingestion, dermal penetration and injection or implantation for biomedical applications [150]. The route of entry, the dose and the duration of nanoparticles into the human body have a significant effect on the extent and severity of the toxicity [16]. The other significant determinants such as dose and duration of exposure also play a vital role in the level of toxicity.

### Impact on the Environment

**Fig. 8:**
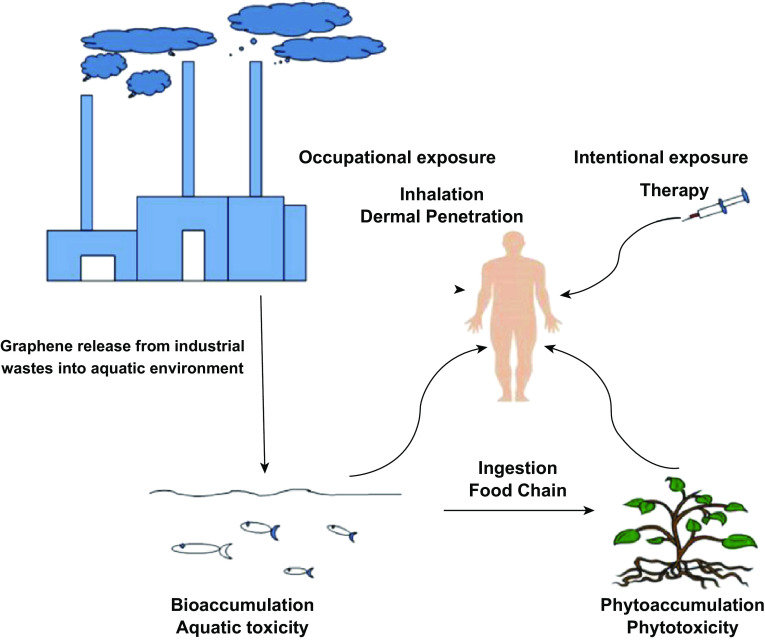
Human exposure to graphene from the environment: Humans are exposed to nanomaterials either intentionally in the form of therapy or unintentionally via various factors especially during the manufacturing process in industries. Graphene can enter ecosystem mainly through waste disposal from industries or pharmaceuticals, posing a threat to aquatic organisms. Stable graphene that exists in soil or water can enter the human body through food chain [151]. Copyright © 2016 Elsevier B.V

Graphene is emerging as a dynamic nanocarbon material. Although there are a broad scope and numerous advantages of GBNs in different fields of the scientific world, they also cause toxic effects on different biological models. An increase in the production of GBNs and their expected usage for biomedical purposes raises anxiety about their effects on humans and environment. It is necessary to understand the interaction of GBNs with the living systems to advance the biomedical application of GBNs. Even though the health effects associated with the GBNs have been studied at the cellular and in animal model, the human exposure of GBNs is unknown. Humans can be affected by GBNs via various exposure routes (Fig. 8) [151] from the site of production to the environment. Thereby, both the abiotic and biotic compartments of the ecosystem will get disturbed. It is imperative to investigate the interaction of GBNs across the membranes in the ecosystem to estimate the risk potential of the GBNs released into the environment. Very few reports found the impact of GBNs on the environment. Among GBNs, GO is considered as toxic. Choudhury et al. and Wu et al. investigated the environmental fate and transport of GO [153–156]. Choudhury et al. investigated the role of sunlight on the physicochemical properties, aggregation and deposition of GO in aquatic environments [155]. They reported that exposure to sunlight has a significant impact on the physiochemical properties of GO and their subsequent transport by reducing the materials stability in the environment. The research needs to be conducted to understand the complex roles of pH, natural organic material and other natural colloids on the fate of photo-transformed GO. Zhao et al. [122] discussed GO transformation to RGO may occur under the direct interaction of aquatic organisms. Hua et al. explored the aggregation and resuspension of GO in simulated natural aquatic environments. The findings indicated that the graphene oxide nanoparticles (GONPs) transport and fate has a significant impact in natural aquatic environments by divalent cations, natural organic matter (NOM) and hydraulics [157].

As it is projected that the GBNs-based products market to reach millions of dollars by 2020, there will be a generation of GBNs-based wastes into the environment. It is essential to evaluate the potential toxic effects and fate of GBNs in the environment. Only a few researchers evaluated the impact of GBNs in environment. Ahmed et al. investigated the acute effects of GO on waste water microbial community [158]. They concluded that GO was toxic to microbial communities in concentrations between 50 and 300 mg L^−1^. The quality of the effluent was deteriorated by increasing the turbidity of water and the reduction in sludge dewaterability. They also confirmed the reactive oxygen species generation is responsible for the toxicity of GO on microbial communities. Deng et al. studied the characterization factors such as toxic effect factor, fate factor and exposure factor of GO in the environment to study the life cycle impact assessment of GO-based nanomaterials [159]. More research has to be conducted as there are very few studies on the environmental risks of GBNs and their strict enforcement on the release of GBNs to mitigate the toxic effects of GBNs. Lee et al. reported their findings on common scenarios (exfoliation, CVD growth and transfer) and the good practices that reduce graphene or GBNs exposure at facilities manufacturing facilities [68]. In addition to toxicity studies, GBNs especially three-dimensional (3D) porous carbon-based materials such as GO and GO-based networks have proven to have potential environmental applications. GBNs were explored for removing organic pollutants to advance in water remediation. Rethinsabapathy et al. summarized 3D GBNs materials used for the adsorption of dyes, heavy metals and radioactive materials from polluted environments [160].

## Conclusions and Perspectives

It is evident that GBNs, because of their unique properties and functionalization, raise a great interest and provide more avenues for the research and development in their applications of translational medicine. The biomedical applications related to the unique physiochemical properties of GBNs focus on their thermal, mechanical and electrochemical features. Many reports have paid attention to GBNs as therapeutics in cancer therapy along with PTT and PDT, gene/drug delivery and as antibacterial agents. The intrinsic optical properties of GBN-based hybrids in the visible and NIR range along with their small size effects, low toxicity and low production costs make the hybrids attractive for bioimaging in clinical diagnostics and photo-thermal cancer therapy. This targeted therapy aids in their high therapeutic effects and fewer side effects. Among GBNs, GO and RGO are considered as the most potent antibacterial agents which can be used in nanohybrids to synthesize novel antibacterial agents. The use of GBNs in bioimaging and biosensing fields is an emerging biomedical application. As the GBNs are used in various fields for biomedical purposes the safety and efficacy of GBNs in clinical trials such as diagnostics and therapeutics require standardized parameters; mainly, biocompatibility, solubility and selectivity are the predominant factors that will further the biomedical applications of GBNs. More studies in computational simulations of GBNs need to be explored for the efficacy of GBNs in clinical trials.

The applications of GBNs have expanded quickly into various fields, but still, there is a lack of systematic understanding of biological interactions of GBNs. The experimental data on the toxicity are limited more to in vitro rather than to in vivo. In addition, there should be more knowledge of the long-term toxicity effects of GBNs to further enhance their applications in the biomedical field to assure the human safety. The existing literature does not provide detailed information on the various synthesis procedures and characterization techniques before proceeding to the toxicological assays. More emphasis should be given on the comprehensive understanding of GBNs-based products on adsorption, dispersion and toxicity, and transformation is recommended. Currently, most of the GBNs are focused on lungs and liver. Studies on other organs including brain/central nervous system are very limited or remain unexplored. Moreover, the GBNs due to excellent physicochemical properties can specifically disrupt the neuroendocrine/reproductive organs. To our knowledge, the reports on GBNs as endocrine disruptor are very limited. Additional studies in these areas are also necessary. Furthermore, GBNs can interact with DNA and thus affect the genetics of human populations. Therefore, studies are needed to elucidate transgenerational effects or effects of GBNs on the epigenome. Reina et al. emphasized the guidelines and the development and evaluation of biocompatibility of GBNs [29]. These guidelines include thorough characterization and regulatory standardization of GBNs, standardized data formats to identify the relationships between structure and properties and finally standard references of known activities of biological tests. Also, more research is required to optimize the synthesis with proper characterization methods to the GBNs with unique properties. The majority of research is on the toxicity at the cellular level rather than the interaction of GBNs at the genetic level. Since other GBNs such as 3D porous graphene materials have attracted great attention for environmental applications in the removal of pollutants of organic, inorganic and radionuclides [160], further studies are needed to evaluate their fate and transport as well as their ecological risks in various environmental compartments. Such research would provide a scientific basis to manage their uses and control/prevent their toxic effects.
